# Chemical Composition, Antimicrobial and Antiparasitic Screening of the Essential Oil from *Phania matricarioides* (Spreng.) Griseb.

**DOI:** 10.3390/molecules24081615

**Published:** 2019-04-24

**Authors:** Yamilet I. Gutiérrez, Ramón Scull, Anabel Villa, Prabodh Satyal, Paul Cos, Lianet Monzote, William N. Setzer

**Affiliations:** 1 Department of Pharmacy, Institute of Pharmacy and Food, Havana University, Coronela, Lisa, Havana 13600, Cuba; ygutierrez@infomed.sld.cu (Y.I.G.); rscull@ifal.uh.cu (R.S.); 2 Genetic toxicology and antitumor laboratory, Drug Research and Development Center (CIDEM), Havana 10600, Cuba; anabel.villa@cidem.cu; 3 Aromatic Plant Research Center, 230 N 1200 E, Suite 100, Lehi, UT 84043, USA; psatyal@aromaticplant.org; 4 Laboratory for Microbiology, Parasitology and Hygiene (LMPH), Faculty of Pharmaceutical, Biomedical and Veterinary Sciences, University of Antwerp, 2610 Wilrijk, Belgium; paul.cos@ua.ac.be; 5 Research Network Natural Products against Neglected Diseases (ResNet NPND); 6 Parasitology Department, Center of Research, Diagnostic and Reference, Institute of Tropical Medicine “Pedro Kouri”, Havana 10400, Cuba; 7 Department of Chemistry, University of Alabama in Huntsville, Huntsville, AL 35899, USA

**Keywords:** *Phania matricarioides*, essential oil, antimicrobial screening, cytotoxicity, *Trypanosoma cruzi*

## Abstract

Essential oils (EOs) have gained increasing attention due to their pharmacological effectiveness, and they also constitute some of the most popular natural products. In this study, we present the chemical characterization of the EO from *Phania matricarioides* and the in vitro activity/selectivity against a wide panel of bacteria, fungi and parasitic protozoa. Forty-five compounds were identified in the studied EO, of which lavandulyl acetate (40.1%) and thymyl isobutyrate (13.9%) were the major components. The EO did not inhibit bacterial or fungal growth at the maximum concentration tested (64 µg/mL), although it displayed activity on all evaluated protozoa (IC_50_ values ranging from 2.2 to 56.6 µg/mL). In parallel, the EO demonstrated a noteworthy cytotoxic activity against peritoneal macrophages (CC_50_ values of 28.0 µg/mL). The most sensitive microorganism was *Trypanosoma cruzi*, which had a superior activity (IC_50_ = 2.2 µg/mL) and selectivity (SI = 13) in respect to other parasitic protozoa and the reference drug (*p* < 0.05). Further in vivo studies are needed to evaluate the potential use of this EO and the main compounds as antitrypanosomal agents. To our knowledge, this is the first report of chemical characterization and antimicrobial assessment of the EO from *P. matricarioides*.

## 1. Introduction

Indiscriminate and irrational use of antimicrobial agents has created an unprecedented challenge for human civilization due to the microbes’ development of antimicrobial resistance. Complementary and alternative medicines (CAMs) are used by 60–80% of developing countries, as they are one of the most prevalent sources of medicine worldwide. In recent years, research on plant-based drugs has increased tremendously, and there is some hope seen in certain medicinal plants that have immense potential to combat bacterial, fungal, protozoal and viral diseases with safety. Such plant metabolites include quinones, alkaloids, lectins, polypeptides, flavones, flavonoids, flavonols, coumarins, terpenoids, phenylpropanoids and tannins [1,2]. In particular, other plant-derived natural products, such as essential oils (EOs), have experienced an upsurge in attention due to their efficacious bioactivities [3], and represent one of the most popular natural product classes due to their applications in dermatology, cosmetics and aromatherapy [4]. There have been numerous publications regarding the utility of EOs as antimicrobial, anticancer, anti-inflammatory and antiviral agents. In recent years, numerous papers reporting antimicrobial properties of EOs extracted from higher plants have been published, including antibacterial, antifungal and antiparasitic activities [5].

**Figure 1:**
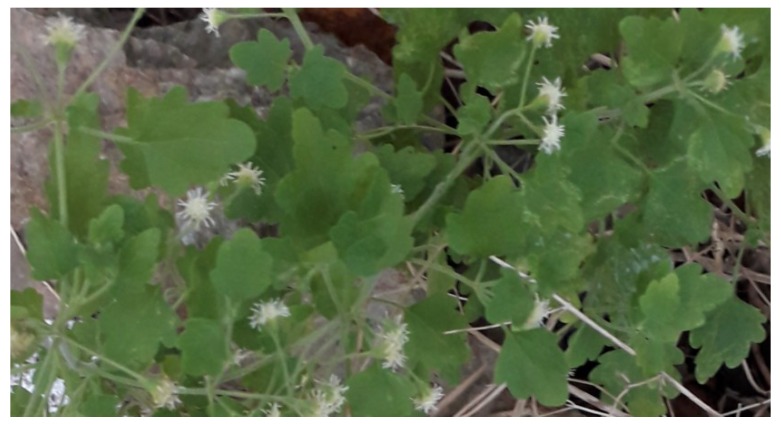
*Phania matricarioides* in a natural habitat. Picture taken by the authors during collection of the plant (March 2013, Bauta municipality, Artemisa, Cuba).

Recently, we have focused on *Phania matricarioides* (Spreng.) Griseb. (Figure 1) (Asteraceae or Compositae family), which has been traditionally used for digestive conditions (stomach pain, bad digestion and diarrhea) and dermatological lesions [6,7]. *P. matricarioides* is included in a very large and widespread family of flowering plants that grow for ornamental purposes [8], but the family also represents a certain economic importance due to the provision of products such as cooking oils, lettuce, sunflower seeds, artichokes, sweetening agents, coffee substitutes and herbal teas [9]. In addition, many species possess medicinal properties, including antioxidant [10], antiparasitic [11], anticancer [12] and hepatoprotective [13]. In particular, the antidiarrheal, analgesic, anti-inflammatory and anti-oxidant effects of extracts have been demonstrated and correlated with some active common phytochemicals such as phenolic compounds, tannins, flavonoids, lactonic compounds, triterpenes or steroids, terpenes and organic acids [6,14,15]. However, as far as we know, the EO from *P. matricarioides* has not been chemically characterized, and its biological properties have not been scientifically demonstrated.

In this study, taking into account the antimicrobial potential of EOs, we have chemically characterized of the EO from *P. matricarioides* and screened for potential activity/selectivity against bacteria, fungi and parasitic protozoa of medical relevance.

## 2. Results

**Figure 2:**
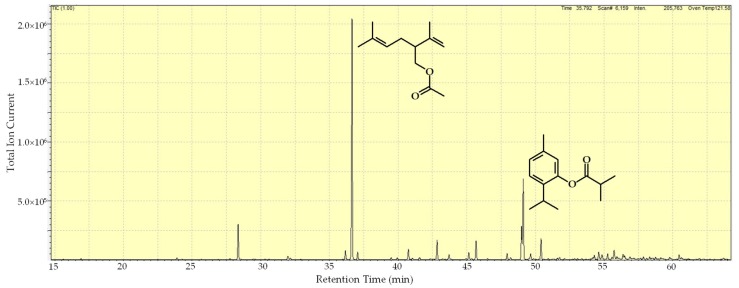
Gas chromatogram and major components of the essential oil extracted by hydrodistillation from *P. matricarioides* collected in Bauta municipality, Artemisa, Cuba.

The obtained volatile oil from *P. matricarioides* was colorless and aromatic, and yielded 0.1 ± 0.002%. The results of the gas chromatography–mass spectrometry (GC/MS) are summarized in Table 1, and show that the chemical composition of the EO was made up of 48 compounds, of which 45 components were identified, representing 98.9% of the total oil. The predominant components were lavandulyl acetate (40.1%), followed by thymyl isobutyrate (13.9%) (Table 1 and Figure 2).

The EO did not exhibit bacterial or fungal growth inhibition at the maximum concentration tested (64 µg/mL). However, as demonstrated in Table 2, the EO displayed activity on all evaluated protozoa with varying degrees of inhibition (IC_50_ values: 2.2–56.6 µg/mL). The most sensitive microorganism was *Trypanosoma cruzi*, which demonstrated a superior susceptibility to the EO (*p* < 0.05) than the other parasitic protozoa, or the reference drug.

In parallel, the EO demonstrated a noteworthy cytotoxic activity against peritoneal macrophages, as shown in Table 2. The most selective parasite was also *T. cruzi*, with a SI greater than 10.

## 3. Discussion

Innovative approaches for effective proof-of-concept research and the development of new types of plant-derived products effective against recently emerging problems related to human microbial diseases are needed. In this regard, research on antimicrobial agents from natural sources has grown in importance in order to discover novel, effective and less expensive drugs, and to combat microbial resistance. Consequently, in our continuing search for valuable and promising natural products from Cuban medicinal plants against infectious diseases, this study was carried out to analyze the chemical composition of the EO from *P. matricarioides*, and was extended to investigate the antimicrobial activity of this product against a wide panel of microorganisms.

Cabrera et al. reported that the yield of EO from fresh aerial parts of *P. matricarioides* range between 0.06% and 0.41% under different conditions of treatment of the plant material (fresh plant material, drying in the shade and drying in a stove) [6]. Thus, in the present investigation, the yield fell into the referred range. Although the oil yield was not high, it played an important role in the biological activities presented by the plant, justifying in some way its traditional dermatological and digestive uses.

In the studied EO, a high percentage of compounds, around 99%, were identified. The chemical composition profile included a broad spectrum of components, such as monoterpene derivatives. The main compound of EO was lavandulyl acetate (acetate ester of lavandulol, a known component of lavender oil), which has been also identified in EOs from *Heracleum sprengelianum* Wight & Arn. [16], *Lavandula angustifolia* Mill. [17] and *Pycnocycla nodflora* Decne. ex Boiss. [18]. The biological potentiality of this compound was demonstrated through the larvicidal activity against three important mosquito vectors [16]. Another major compound was thymyl isobutyrate, a phenolic monoterpene derivative (ester of thymol) with recognized antimicrobial activity [19].

EOs improve the shelf-life of packaged products and control microbial growth [20], which has provided evidence that EOs are natural antimicrobials isolated from plants [21]. There have been no previous studies on the antimicrobial activity of *P. matricarioides*, and our results showed that the tested bacteria and fungi strains were not sensitive to the studied oil at the concentrations tested.

There have been several studies reported in the literature, supporting the suggestion that certain EOs from various plant species can demonstrate antiparasitic activity [22,23,24,25]. As has been demonstrated in the present study, the EO from *P. matricarioides* inhibited the growth of all tested parasites, with IC_50_ ranging from 2 to 57 µg/mL. In comparison with reference drugs, the studied EO showed higher IC_50_ values with statistical differences (*p* < 0.05), except in the case of *T. cruzi*, which displayed higher sensitivity (*p* < 0.05) to the EO than to benznidazole. Other Cuban EOs, have also demonstrated a wide spectrum of antiprotozoal activity, including *Chenopodium ambrosioides* L. [22] and *Piper aduncum* L. [23]. Nevertheless, as biological properties of the main identified compounds in the studied EO are still scarce, further studies of pure compounds, other components and/or synergistic effects remain to be analyzed to explain the observed activities.

Meanwhile, cytotoxic effects on macrophages from mice were determined and used to calculate the selectivity index (SI). The EO showed low SI values in general, except in the case of *T. cruzi*. It is known that one of most important criteria to select “hits” during in vitro screening is to demonstrate a SI superior to 10 [26], which was only accomplished by the *T. cruzi* parasite (SI = 13).

*T. cruzi,* the causal agent of Chagas disease, is a complex zoonosis transmitted by more than 100 species of triatomine insects (Hemiptera: Reduviidae: Triatominae) and sustained by more than 70 genera of mammalian hosts, including humans [27]. Infection is endemic from Northern Mexico to Argentina, and is considered the parasitic infection with the greatest socioeconomic burden in Latin America, although human migrations have earned it global importance [28,29]. Clinical development of the disease has two successive phases: acute phase (typically asymptomatic or with variable symptoms that may last four to eight weeks) and chronic phase (presented in the majority of patients as chronic infection, which can develop irreversible lesions of the heart, esophagus or colon, ultimately leading to death of the patient) [27,30]. Although many vector control programs have been implemented, *T. cruzi* has not been eradicated, and the development of an effective human vaccine against Chagas disease has been slow [31]. Currently, the only two anti-trypanosomal drugs available for Chagas disease—benznidazole and nifurtimox—are far from ideal. Both require prolonged treatment, display a wide range of side effects and, although being effective in the acute phase, have a limited efficacy in the chronic stage [32,33]. In this sense, one approach to the development process of new antitrypanosomal drugs has been the search of natural products [34]. In particular, several EOs from plants, such as *Alpinia speciosa* K. Schum [25], *Lippia alba* N.E.Br. ex Britton & P. Wilson [35], *Piper tuberculatum* Jacq. [24] and *Protium ovatum* Engl. [36], have demonstrated efficacy against *T. cruzi*.

Against the other tested protozoal parasites, higher IC_50_ values were obtained (7.5–56.6 µg/mL). However, in this case it should be taken into account that EOs are mixtures of different components, and the main antiprotozoal principle could be in a lower concentration. As a consequence for these parasites, lower SI values (≤ 4) were also observed, which limit the potential application of the studied EO by a systemic administration route, although the application by a topical route could be feasible as well as the use of carriers such as nanoparticles [37]. In particular, the current search for new alternatives against *P. falciparum, L. amazonensis* and *L. infantum* (causal agents of malaria, cutaneous and visceral leishmaniasis, respectively) constitute an interesting approach to target multiparasite infections with the same drugs in the control programs of endemic countries [38].

## 4. Materials and Methods

### 4.1. Plant Material

The aerial parts of *P. matricarioides* were collected early in the morning in March 2013 in Bauta municipality (22°59′31″ N, 82°32′57″ W, 10 m asl), Artemisa province, Cuba. A plant specimen was authenticated by Professor Jorge Gutiérrez Amaro and deposited in the Herbarium of National Botany Garden of Havana, Cuba, under the voucher number HFC 88669. Vegetable material was washed with abundant common water and manually crushed.

### 4.2. Essential Oil Extraction and Chemical Characterization

To obtain the EO from *P. matricarioides*, fresh vegetal material was hydrodistilled using a Clevenger type apparatus for 5 h. For the biological assays, the EO was dissolved in dimethyl sulfoxide (DMSO) at 20 mg/mL.

Chemical characterization of the EO was carried out by gas chromatography coupled with a mass spectrometric detector (GC–MS) using Shimadzu GCMS-QP2010 Ultra (Shimadzu Scientific Instruments, Columbia, MD, USA) equipment, which was operated in the electron impact (EI) mode (electron energy = 70 eV), with a scan range = 40–400 atomic mass units, scan rate = 3.0 scans/s and GC-MS solution software. The GC column was a ZB-5-fused silica capillary column with a (5% phenyl)-polymethylsiloxane stationary phase and a film thickness of 0.25 μm, a length of 30 m and an internal diameter of 0.25 mm (Phenomenex, Torrance, CA, USA). The carrier gas was helium with a column head pressure of 552 kPa and a flow rate of 1.37 mL/min. The injector temperature was 250 °C and the ion source temperature was 200 °C. The GC oven temperature program was programmed for a 50 °C initial temperature, with temperature increased at a rate of 2 °C/min to 260 °C. A 5% *w/v* solution of the sample in CH_2_Cl_2_ was prepared, and 0.1 μL was injected with a splitting mode (30:1). Identification of the oil components was based on their retention indices (RI) as determined in reference to a homologous series of *n* alkanes using the method of Kovats [39], and by comparison of their mass spectral fragmentation patterns with those reported in the literature [40], and stored in our in-house Sat-Set library [41].

### 4.3. Microorganisms

An integrated panel of microbial agents in a 96-well plate was adopted from Cos et al. [42], including Gram-negative *Escherichia coli* (ATCC8739), Gram-positive *Staphylococcus aureus* (ATCC6538), yeast *Candida albicans* (B59630), and protozoa *Plasmodium falciparum* (Ghana), *Trypanosoma brucei brucei* (Squib-427), *T. cruzi* (Tulahuen CL2), *L. infantum* (MHOM/MA(BE)/67) and *Leishmania amazonensis* (MHOM/77BR/LTB0016).

### 4.4. Cell Culture

Cytotoxicity was tested against peritoneal macrophage from BALB/c mice (PMM) obtained by peritoneal washing in RPMI medium (Sigma, St. Louis, MO, USA) and antibiotics (100 μg of streptomycin/mL, 100 U of penicillin/mL; Sigma, St. Louis, MO, USA) at the moment of use.

### 4.5. Antibacterial and Antifungal Assays

*E. coli* and *S. aureus* were cultured in Mueller Hinton broth (MHB; Sigma-Aldrich, St. Louis, MO, USA) medium; while *C. albicans* was cultured in RPMI medium. In all cases, 5 × 10^3^ CFU/well was added with different EO concentrations ranging from 0.25 to 64 µg/mL. After 17 h of incubation at 37 °C, bacterial or fungal viability was determined fluorimetrically by the addition of resazurin (Sigma-Aldrich, St. Louis, MO, USA) for 30 min at 37 °C to bacteria cultures, or 4 h at 37 °C to fungi [43]. Finally, fluorescence was measured using a Tecan GENios Multifunction Fluorimeter (Tecan Group, Maennedorf, Switzerland) at 530 nm excitation and emission of 590 nm. At the same time, reference drugs were tested, including chloramphenicol (Sigma-Aldrich, Bornem, Belgium) for *E. coli*, erythromycin (Sigma-Aldrich, Bornem, Belgium) for *S. aureus* and miconazole (Janssen Pharmaceuticals, Beerse, Belgium) for *C. albicans*.

### 4.6. Antiprotozoal Assays

Antiplasmodial activity was determined with parasites cultured in human erythrocytes A+ in RPMI-1640 culture medium, supplemented with 0.5% (*w*/*v*) Albumax at 37 °C under an atmosphere of 3% O_2_, 4% CO_2_ and 93% N_2_ [44]. Suspensions of infected human red blood cells (1% parasitemia, 2% hematocrit) were added to each well with the same test concentrations of EO. The plate was incubated for 72 h under the same conditions, and then the plate was frozen at −20 °C. Parasite multiplication was measured after mixing 20 μL of the hemolyzed parasite suspension with 100 µL of Malstat (Flow Incorporated, USA) reagent in a new plate, and incubated for 15 min at room temperature. After that, 20 μL of nitro blue tetrazolium chloride (NBT; Sigma Aldrich, St. Louis, MO, USA) at 2 mg/mL/phenazine ethosulfate (PES; Sigma Aldrich, St. Louis, MO, USA) at 0.1 mg/mL solution was added. The plate was incubated again for 2 h at room temperature in the dark and the absorbance was read at 655 nm in a Biorad 3550-UV microplate reader. Chloroquine, donated by the Special Programme for Research and Training in Tropical Diseases from the World Health Organization (WHO-TDR), was also included as a reference drug.

Antitrypanosomal activity was performed using trypomastigotes of *T. brucei*, cultured in Hirumi-9 medium supplemented with 10% inactivated fetal calf serum (FCSi) at 37 °C and 5% CO_2_ [45]. Assays were performed by adding 1.5 × 10^4^ trypomastigotes/well to the EO at the mentioned concentrations. After 72 h of incubation at the same conditions, parasite growth was assessed fluorimetrically by adding resazurin for 24 h at 37 °C. In parallel, activity on amastigotes of *T. cruzi* was also evaluated. In this case, the EO at tested concentrations was added to 4 × 10^4^ amastigotes in 4 × 10^3^ MRC-5 cells maintained in minimal essential medium (MEM; Life Technologies, USA) supplemented with 20 mM L-glutamine, 16.5 mM sodium bicarbonate and 5% of FCSi. After an additional incubation for seven days at the previous conditions, parasite growth was assessed by adding the β-galactosidase substrate chlorophenol red β-D-galactopyranoside (Sigma Aldrich, St. Louis, MO, USA) and then incubating for 4 h at 37 °C. The absorbance was then read at 540 nm [46]. As reference drugs, suramine for *T. brucei* and benznidazol for *T. cruzi* were used, which were kindly donated by WHO-TDR.

Antileishmanial activities against the intracellular amastigote form of *L. infantum* and *L. amazonensis* were performed. For *L. infantum*, 3 × 10^4^ PMM were infected with amastigotes obtained from an infected hamster at a density of 15 parasites per cell, and the plate was incubated for 48 h at 37 °C and 5% CO_2_. Pre-diluted concentrations of EO were added and the plates were then incubated under the same conditions over a 120-h period. In the experiment with *L. amazonensis*, PMM were plated at 10^6^/mL in a 24-well Lab-Tek (Costar, USA) and incubated at 37 °C and 5% CO_2_ for 2 h. Non-adherent cells were removed and stationary-phase promastigotes were added at a 4:1 parasite/macrophage ratio for 4 h. Cells were washed to remove free parasites, EO was added, four serial dilutions were carried out to test from 12.5 to 100 µg/mL and the plates were further incubated at same conditions for 48 h [47]. In both cases, after the incubation period with the products, the supernatant was discarded and cells were fixed with methanol, stained with 10% Giemsa and microscopically examined (Motic, Japan) under immersion oil. The total parasite burden was determined according to the number of infected macrophages and the number of amastigotes inside the macrophages. Miltefosine (donated by WHO-TDR) and amphotericin B (Imefa, La Habana, Cuba) were used as reference drugs, respectively.

### 4.7. Cytotoxicity Assay

To assess the cytotoxic effects of the EO, isolated PMM were seeded at 3 × 10^5^ cells/mL and incubated at 37 °C and 5% CO_2_ [48]. After 2 h, the medium was removed and 98 µL of fresh medium with 10% FCSi and antibiotics were added, with an additional 48 µL in the first wells and 2 µL of pre-diluted EO, ranging from 12.5 to 200 µg/mL. The plate was incubated at same conditions for 48 h, and viability was measured with 15 μL of 3-[4,5-dimethylthiazol-2-yl]- 2,5-diphenyltetrazolium bromide (MTT; SIGMA, St. Louis, MO, USA) solutions. After 4 h, formazan crystals were dissolved with 100 μL of DMSO and the optical density was measured at 560 nm and at 630 nm as a reference wavelength using a spectrophotometer (Molecular Devices, Silicon Valley, California, USA).

### 4.8. Statistical Analysis

In each case, percentage growth inhibition for each concentration of EO was calculated compared to the untreated controls. The median inhibitory concentration (IC_50_) for antibacterial, antifungal and antiprotozoal assay were determined, while median cytotoxic concentrations (CC_50_) were obtained in the cytotoxicity experiment. In each case, the values were calculated from lineal dose-response curves, and results are expressed as the means of three experiments. To compare the IC_50_ and CC_50_ values between EO and reference drugs, statistical differences were determined using Mann-Whitney with Statistica for Windows program (Release 4.5, StatSoft, Inc., Tulsa, OK, USA, 1993), considering statistical differences as *p* < 0.05. Finally, the selectivity index (SI) was calculated for each evaluated product, which was determined by CC_50_/IC_50_.

## 5. Conclusions

To our knowledge this work is the first investigation related to the EO from *P. matricarioides*, and confirms the importance of chemical, biological and cytotoxicity assessments of EOs. The results demonstrate the wide spectrum of antiparasitic activities, in particular against *T. cruzi*, with a potential margin of safety based on the high SI obtained from comparison with mammalian cells. Further in vivo studies are needed to evaluate the potential use of this EO and its main compounds as antitrypanosomal agents.

## Figures and Tables

**Table 1:** Peak assignment for the gas chromatography–mass spectrometry profile of the essential oil extracted by hydrodistillation from *Phania matricarioides* collected in Bauta municipality, Artemisa, Cuba.

RI ^a^	Compound	Area (%)
994	Yomogi alcohol	0.1
1099	Linalool	0.3
1154	(*Z*)-chrysenthemol	0.2
1163	Lavandulol	5.5
1215	Coahuilensol methyl ether	0.7
1217	3-isopropylbenzaldehyde	0.2
1276	Chrysanthemyl acetate	1.4
1283	Lavandulyl acetate	40.1
1289	Thymol	1.2
1325	Silphiperfol-5-ene	0.3
1332	δ-elemene	0.3
1344	7-*epi*-silphiperfol-5-ene	1.8
1349	Neryl acetate	0.2
1357	Silphiperfol-4,7(14)-diene	0.4
1376	α-copaene	3.2
1389	β-elemene	0.9
1411	Thymohydroquinone dimethyl ether	1.2
1415	Lavandulyl isobutyrate	0.1
1420	β-caryophyllene	3.3
1456	α-humulene	1.1
1460	*allo*-aromadendrene	0.3
1473	8,9-dehydrothymyl isobutyrate	5.9
1475	Thymyl isobutyrate	13.9
1483	Neryl isobutyrate	1.0
1495	Bicyclogermacrene	3.7
1515	Cubebol	0.3
1518	β-guaiene	0.4
1536	Silphiperfol-5-en-3-ol B	0.2
1545	Silphiperfol-5-en-3-one B	0.1
1558	Silphiperfol-5-en-3-ol A	0.3
1560	(*E*)-nerolidol	0.7
1565	Thymyl 2-methylbutanoate	1.6
1570	Neryl 2-methylbutanoate	1.1
1576	Spathulenol	0.9
1582	Caryophyllene oxide	0.4
1584	*allo*-spathulenol	1.7
1588	β-copaen-4α-ol	0.3
1595	Fokienol	0.8
1604	Ledol	0.4
1618	Silphiperfol-6-en-5-one	0.2
1621	Unidentified ^b^	0.5
1629	Unidentified ^c^	0.4
1632	Caryophylla-4(12),8(13)-dien-5α-ol	0.5
1637	Caryophylla-4(12),8(13)-dien-5β-ol	0.4
1644	τ-murrolol	0.2
1655	α-cadinol	0.3
1667	6-methoxythymyl isobutyrate	0.9
1726	Unidentified ^d^	0.2
	TOTAL IDENTIFIED = 98.9%	

^a^ RI: retention index (determined with respect to a homologous series of *n* alkanes on a ZB-5 column). ^b^ MS(EI): 218(24%), 203(8%), 175(36%), 161(17%), 147(59%), 133(46%), 120(53%), 119(80%), 107(74%), 105(100%), 93(42%), 91(60%), 79(37%), 69(29%), 55(37%), 43(36%), 41(37%). ^c^ MS(EI): 164(4%), 146(69%), 135(100%), 115(10%), 91(16%), 71(28%), 43(70%). ^d^ MS(EI): 149(5%), 135(3%), 121(5%), 107(3%), 93(8%), 83(100%), 68(9%), 55(24%), 43(5%).

**Table 2:** Antiprotozoal activity and cytotoxic effects of essential oil extracted by hydrodistillation from *P. matricarioides* collected in Bauta municipality, Artemisa, Cuba.

	IC_50_ (µg/mL) ^a^				
	*P. falciparum*	*T. brucei*	*T. cruzi*	*L. amazonensis*	*L. infantum*
Essential oil	20.7	8.0	2.2	56.6	7.5
Reference drug ^c^	0.02	0.04	3.2	0.02	3.7
	**CC_50_ (µg/mL) ^b^**				
	**Peritoneal Macrophage**				
Essential oil	28.0				
Reference drug ^c^	5.8				
	**Selectivity Index ^d^**				
	***P. falciparum***	***T. brucei***	***T. cruzi***	***L. amazonensis***	***L. infantum***
Essential oil	1	4	13	0	4
Reference drug ^c^	290	145	2	290	2

^a^ IC_50_: Median inhibitory concentration. ^b^ CC_50_: Median cytotoxic concentration. ^c^ Reference drugs: Chloroquine for *Plasmodium falciparum*, suramine for *Trypanosoma brucei*, benznidazol for *Trypanosoma cruzi*, miltefosine for *Leishmania infantum* and amphotericin B for *Leishmania amazonensis* and peritoneal macrophage. ^d^ Selectivity index (SI): CC_50/_IC_50_.

## Abbreviations

CAMs: complementary and alternative medicines; CC_50_: median cytotoxic concentration; DMSO: dimethyl sulfoxide; EOs: essential oils; FCSi: inactivated fetal calf serum; IC_50_: median inhibitory concentration; MEM: minimal essential medium; MHB: Mueller Hinton Broth medium; MTT: 3-[4,5-dimethylthiazol-2-yl]-2,5-diphenyltetrazolium bromide; NBT: nitro blue tetrazolium chloride; PES: phenazine ethosulfate; PMM: peritoneal macrophage from BALB/c mice; RI: retention index; SI: selectivity index; WHO-TDR: Special Programme for Research and Training in Tropical Diseases from the World Health Organization.
